# SNP Haplotype Mapping in a Small ALS Family

**DOI:** 10.1371/journal.pone.0005687

**Published:** 2009-05-25

**Authors:** Katherine A. Dick Krueger, Shoji Tsuji, Yoko Fukuda, Yuji Takahashi, Jun Goto, Jun Mitsui, Hiroyuki Ishiura, Joline C. Dalton, Michael B. Miller, John W. Day, Laura P. W. Ranum

**Affiliations:** 1 Departments of Genetics, Cell Biology and Development, University of Minnesota, Minneapolis, Minnesota, United States of America; 2 Institute of Human Genetics, University of Minnesota, Minneapolis, Minnesota, United States of America; 3 Epidemiology and Community Health, University of Minnesota, Minneapolis, Minnesota, United States of America; 4 Department of Neurology, University of Minnesota, Minneapolis, Minnesota, United States of America; 5 Department of Neurology, The University of Tokyo, Tokyo, Japan; Columbia University, United States of America

## Abstract

The identification of genes for monogenic disorders has proven to be highly effective for understanding disease mechanisms, pathways and gene function in humans. Nevertheless, while thousands of Mendelian disorders have not yet been mapped there has been a trend away from studying single-gene disorders. In part, this is due to the fact that many of the remaining single-gene families are not large enough to map the disease locus to a single site in the genome. New tools and approaches are needed to allow researchers to effectively tap into this genetic gold-mine. Towards this goal, we have used haploid cell lines to experimentally validate the use of high-density single nucleotide polymorphism (SNP) arrays to define genome-wide haplotypes and candidate regions, using a small amyotrophic lateral sclerosis (ALS) family as a prototype. Specifically, we used haploid-cell lines to determine if high-density SNP arrays accurately predict haplotypes across entire chromosomes and show that haplotype information significantly enhances the genetic information in small families. Panels of haploid-cell lines were generated and a 5 centimorgan (cM) short tandem repeat polymorphism (STRP) genome scan was performed. Experimentally derived haplotypes for entire chromosomes were used to directly identify regions of the genome identical-by-descent in 5 affected individuals. Comparisons between experimentally determined and *in silico* haplotypes predicted from SNP arrays demonstrate that SNP analysis of diploid DNA accurately predicted chromosomal haplotypes. These methods precisely identified 12 candidate intervals, which are shared by all 5 affected individuals. Our study illustrates how genetic information can be maximized using readily available tools as a first step in mapping single-gene disorders in small families.

## Introduction

The identification of genes for Mendelian disorders has been a highly effective approach for understanding disease mechanisms and normal gene function [1], [2]. One of many examples is the identification of dystrophin gene as the cause of Duchenne muscular dystrophy. This initial discovery led investigators to uncover additional disease genes that cause various forms of muscular dystrophy by affecting the structure and function of distinct proteins within the dystrophin-dystroglycan complex [3]. Additionally, single-gene discoveries have also been instrumental in shedding light on multigenic and sporadic disorders. For example, *adenomatous polyposis coli* (*APC*) mutations in hereditary colon cancer directly led to the identification of other genes involved in the more common sporadic and polygenic forms of colon cancer [4]. It is currently estimated that genes for approximately 3,705 Mendelian and suspected Mendelian disorders have not yet been mapped [5] and because many familial disorders have not yet been formally described in the literature, the number of unidentified single-gene disorders is likely to be grossly underestimated [2]. Although the identification of the causes of these disorders will almost certainly have broad and significant impact on our understanding of the pathophysiology underlying major disease classes (e.g. neurodegenerative, cancer and heart disease), there has been a trend away from studying single gene disorders, in favor of the more common complex diseases. This is in part due to the fact that many of the remaining single-gene families are difficult to study because they have rare mutations and/or are not large enough to map the disease locus to a single site in the genome. New tools and approaches are needed to allow researchers to effectively utilize these important families. Towards this goal, we have used haploid cell lines to experimentally validate the use of high-density SNP arrays to define genome wide haplotypes and candidate regions, using a small ALS family.

Clinically, typical ALS is a neurodegenerative disease selectively involving upper and lower motor neurons that progresses from initial symptoms to death in three to five years. The etiology of most cases of ALS remains obscure, with proposed mechanisms including viral, autoimmune, excitotoxic, metabolic/mitochondrial, toxic or apoptotic processes, protein misfolding or altered axonal transport [6]–[9]. There is, however, no conclusive evidence that any of these pathways are responsible for even a small fraction of ALS cases.

Although ALS usually occurs sporadically, without family history (sALS), ∼10% of cases are familial (fALS), generally with an autosomal-dominant pattern of inheritance [9], [10]. While fALS is uncommon, investigation of families with this disease have provided significant insight into the causes of ALS. The first example was the discovery that mutations in the *Cu/Zn superoxide dismutase (SOD1)* gene cause ALS1 [11], which is thought to account for 20–25% of fALS and 1–3% of sALS cases [9], [12]–[14]. Additional genes that cause dominantly inherited forms of clinically typical ALS include the *vesicle associated membrane protein (VAMP)-associated protein B (VAPB)* (ALS8) [15], [16], the *TAR DNA binding protein (TARDBP)* [17] and the *fused in sarcoma/translated in liposarcoma (FUS/TLS)* [18], [19].

**Figure 1:**
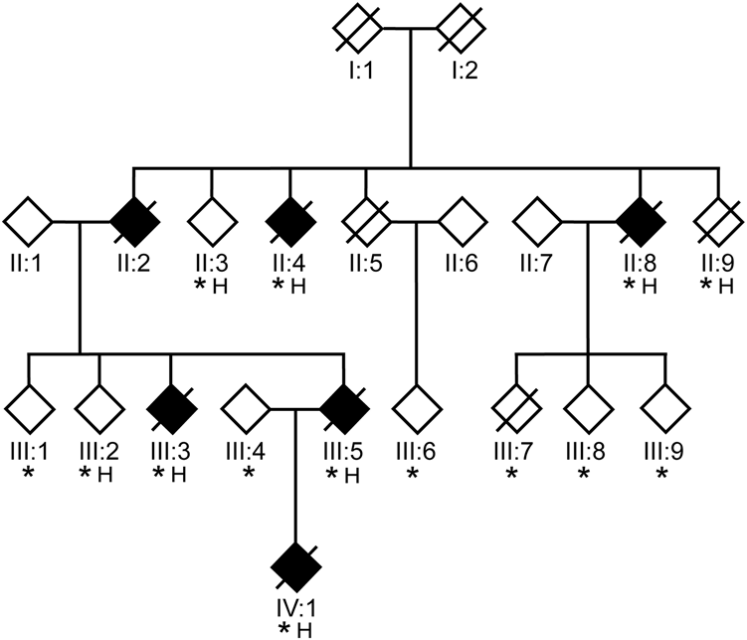
ALS-A Pedigree. Affected individuals are indicated with solid symbols and symbols with a line show the individual is deceased. The asterisk indicates individuals from whom DNA was collected and the H denotes individuals from whom haploid cell lines were generated. For confidentiality purposes gender is not indicated.

While these discoveries have been important for increasing our understanding of the causes of ALS and for developing and testing various treatment strategies, our understanding of the molecular underpinnings of ALS is still in its infancy and identifying additional mutations with forms of ALS that are clinically similar to sALS is likely to clarify the molecular pathways involved in these diseases. However, large families with dominantly inherited ALS are difficult to study because the lethality of the disease limits the ability to obtain DNA from affected individuals. Furthermore, family members can be reluctant to participate in research studies because they do not want to consider the possibility that they or their children might be at risk. For these reasons, the novel ALS family (ALS-A) we have been studying for the past 19 years is of significant scientific importance (Figure 1). We have collected blood from 14 members of this family, including 5 affected individuals, and as a first step in positional cloning have used haploid and high-density SNPs analysis to precisely define all of the regions of the genome that are shared among affected individuals. Because the disorder in the ALS-A family is indistinguishable from sALS, the identification of the genetic cause of this disorder is likely to provide insight into the pathogenic mechanisms of the more common sporadic disease which may ultimately lead to more effective treatments.

## Results

### The ALS-A Family

A pedigree of the ALS-A family is shown in Figure 1. The disease in this family is phenotypically indistinguishable from sALS, and characterized by progressive upper and lower motor neuron degeneration without the involvement of sensory nerves or other complex neurological features, such as frontotemporal dementia (FTD) or Parkinson's features. Age of onset varies, ranging from 35 to 73 years. Lifespan after initial diagnosis ranged from 6 months to 5 years; the individual who lived for five years had a tracheotomy and mechanical ventilation for approximately one year. Simulated two point linkage analysis predicts a maximum logarithm of the odds (LOD) score for the family of 3.17 at Θ = 0.00. The dominant inheritance pattern and number of meioses predict that ∼3% of the diploid genome plus the mutation is likely to be shared among affected family members (0.5^∧5^). Conversely, if completely informative, unambiguously defined haplotypes should allow ∼97% of the genome which is not shared by the affected individuals to be excluded. A limitation of traditional linkage analysis, which is illustrated by the results of an 777 STRP marker screen and conventional multipoint analysis on the ALS-A family, is that there is a significant disparity between the theoretical portion of the genome that should be excluded from containing the ALS-A locus (>97%), and the portion of the genome actually excluded (67%). This disparity results because the markers are not fully informative and haplotypes can not be distinguished.

### Haplotype Analysis: STRP Markers and Haploid DNA

Although haplotype analysis is often used to precisely follow the segregation of small chromosomal regions, recombination and uninformative markers have historically made establishing unequivocal haplotypes for large chromosomal regions impossible. To test if obtaining accurate chromosomal haplotypes can be achieved using high-density SNP arrays and if this would provide more complete segregation information, we directly compared high-density SNP genotype analysis with experimentally determined haplotypes ascertained using a panel of haploid mouse-human hybrid cell lines generated from ALS-A family members [20].

**Figure 2:**
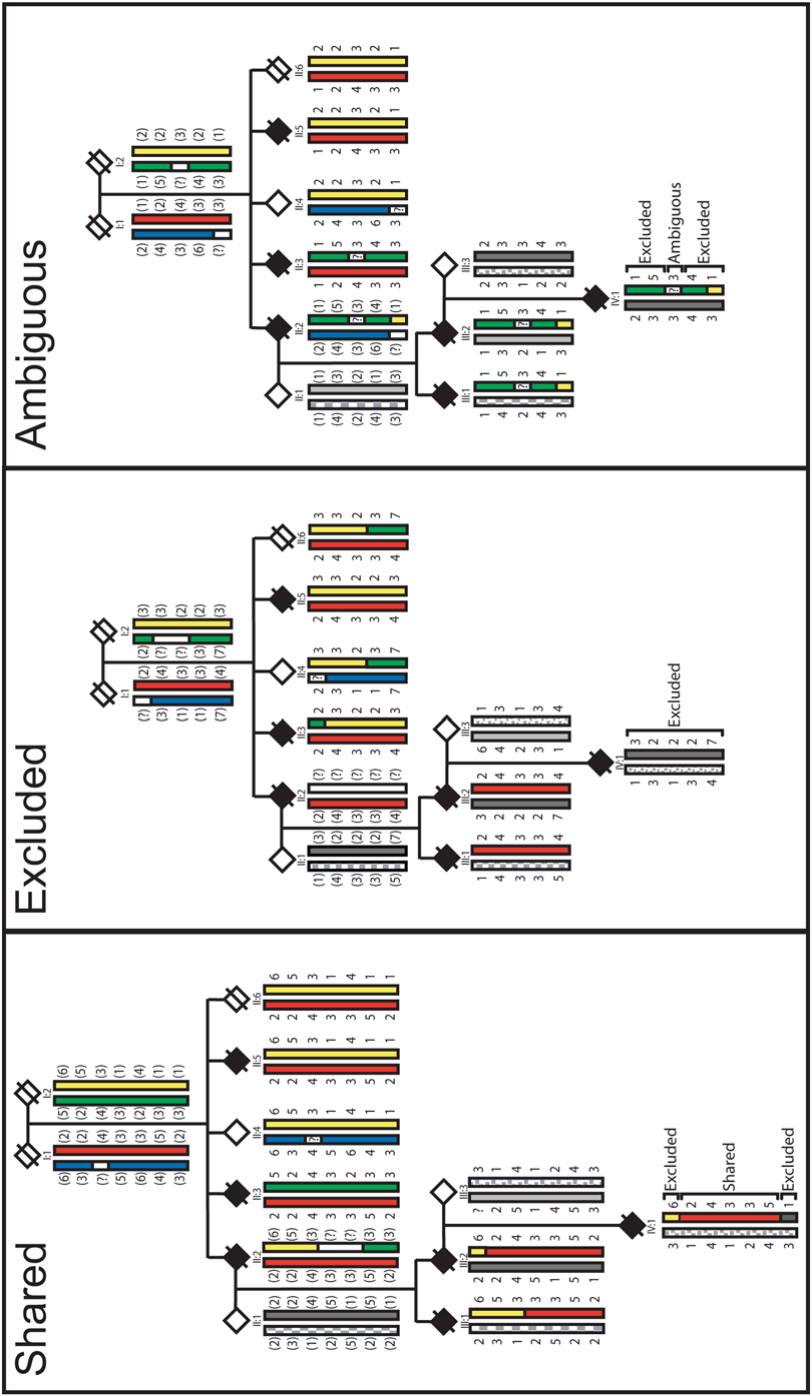
Examples of the Haplotype Analysis. Haplotypes from the founder generation (I) were reconstructed using haplotypes defined by haploid analysis of generation II (colored either red or blue and yellow or green). In subsequent generations the chromosomal regions inherited from unrelated members were identified and eliminated from the analysis (variations of grey). Regions that are shared among the affecteds, excluded, or ambiguous were then directly determined. Where a marker was not fully informative, the founder haplotype was not designated and the region was subsequently denoted by a question mark. Data presented is from chromosome 17.

Experimental haplotypes were established by generating panels of 18–37 haploid cell lines for eight family members including five affecteds. A 777 STRP marker genome scan was performed on DNA from the haploid cell lines, as well as diploid lymphocyte DNA. Genotyping results from chromosome separated cell lines were used to directly define haplotypes for entire chromosomes. Figure 2 illustrates how haplotype comparisons were used to identify shared, excluded and ambiguous regions using chromosome 17 as an example. Because DNA was not available for individuals I∶1 or I∶2, transmitted haplotypes were arbitrarily assigned to the parental generation by genotyping chromosome separated cell lines from a single affected individual in generation II. Specifically, one of the parental chromosomal haplotypes from generation I was designated by a RED bar and the haplotype transmitted from the other parent was assigned a YELLOW bar. Recombinant haplotypes defined by the haploid cell lines of other members of generation II were used to predict the other two founder haplotypes (BLUE and GREEN). Haplotypes from spouses who married into the family and do not contain the ALS-A mutation are indicated by grey bars. STRP markers were spaced at ∼5 cM intervals and double recombinations were expected to be infrequent. Genomic intervals that are found among all five affected individuals are indicated by a shared chromosomal region of the same color (RED or YELLOW). Because any of the regions of the genome that are shared (i.e. identical by descent) among the affected individuals could contain the mutation, all shared regions are considered candidates. Areas not shared among all five affected individuals are excluded, while regions are considered ambiguous if the probability of the double recombination was greater than 1/100 (the threshold for exclusion by LOD score analysis) (Figure 2). Diploid genotypes from 777 STRP markers were also analyzed using the parametric multipoint linkage program VITESSE [21].

### Comparison of STRP Haplotype and LOD Score Analyses

**Figure 3:**
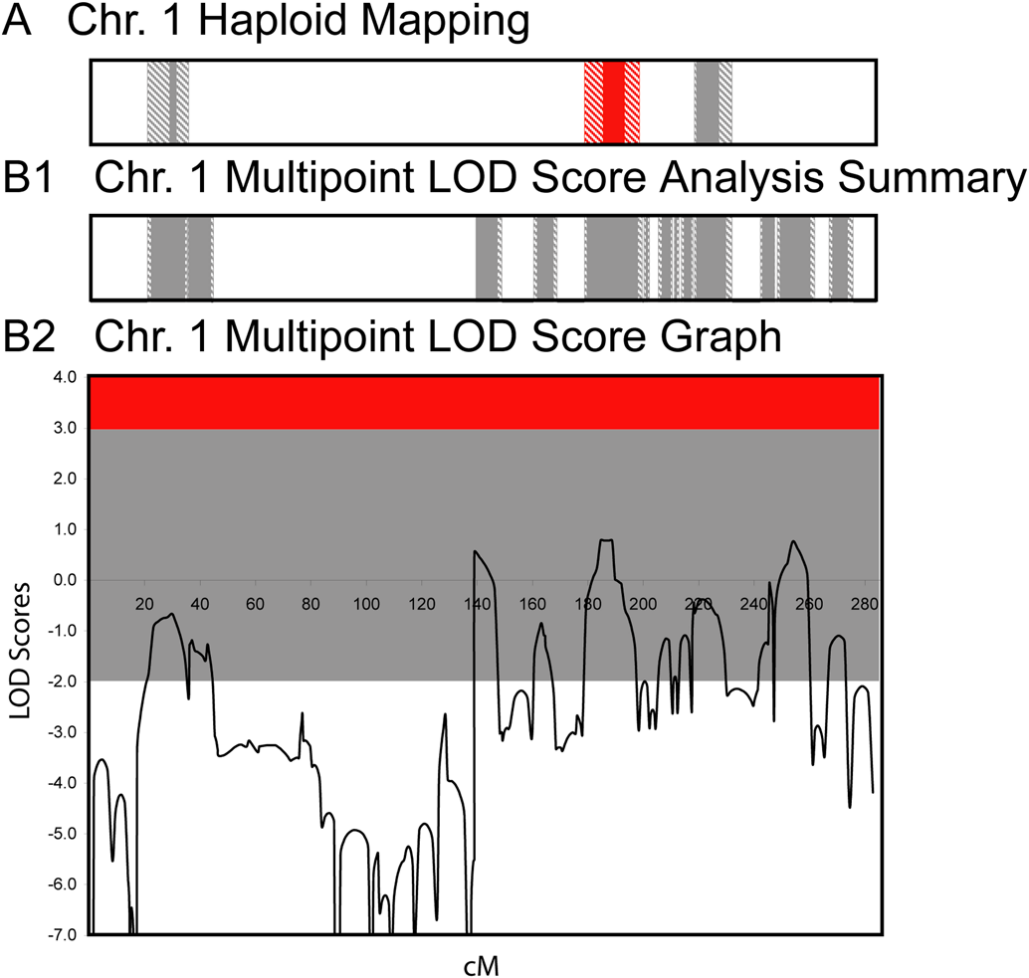
Chromosome 1 Comparison of Excluded, Shared or Ambiguous Regions for Haploid Mapping vs. Multipoint Linkage Analysis. Comparison of information obtained from haploid mapping (A) and multipoint linkage analysis of diploid DNA (B) for chromosome 1. Summaries of the LOD scores are presented in B1 and graphs of the actual LOD scores are illustrated in B2. Regions excluded by haploid mapping or multipoint linkage analysis (LOD<−2.0) are white and ambiguous regions are grey (LOD scores between −2 and +3). Shared regions defined as identical by descent through haploid mapping or with a LOD score >3.0 are shown in red.

**Figure 4:**
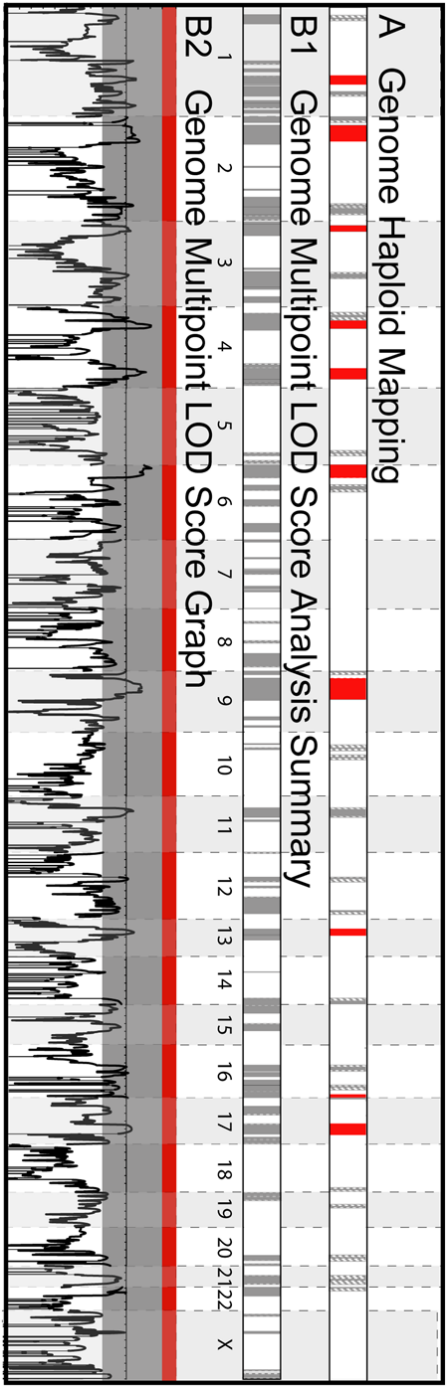
Genome Comparison of Excluded, Shared or Ambiguous Regions for Haploid Mapping vs. Multipoint Linkage Analysis. Comparison of the information obtained from haploid mapping (A) and multipoint linkage analysis (B) for the entire genome. Summaries of the LOD scores are presented in B1 and graphs of the actual LOD scores are illustrated in B2. Regions excluded by haploid mapping or multipoint linkage analysis (LOD<−2.0) are white and ambiguous regions are grey (LOD scores between −2 and +3). Shared regions defined as identical by descent through haploid mapping or with a LOD score >3.0 are shown in red.

Figures 3 and 4 show a comparison of the effectiveness of multipoint linkage using STRP markers and haploid mapping to define shared or excluded regions for chromosome 1 and across the entire genome, respectively. Specifically, three positive LOD scores were generated for chromosome 1, which correspond to a shared region (LOD = 0.785) and two regions known to be excluded by haplotype analysis (LOD = 0.557 & 0.76) (Figure 3). This comparison illustrates the problems investigators face when using small families for linkage analysis —shared and excluded regions are not accurately defined and candidate regions are not always easily distinguished. Genome-wide, haploid mapping identified 10 regions (7.4% of the genome) identical by descent or shared among the 5 affected individuals and definitively excluded 83.1% of the genome as unshared (Figure 4). In contrast, traditional multipoint LOD score analysis using STRP markers on diploid DNA excluded only 67.1% (LOD<−2), failed to identify any shared regions (LOD>3), and generated suggestive scores (most between 0.5–1.2) for regions that were both shared and definitively excluded by haploid analysis. In addition to the shared regions, haploid mapping identified 24 ambiguous regions (indicated in grey), which most likely result from an uninformative marker. The use of haploid cell lines increased the amount of the genome that could be excluded (83%) in comparison to the traditional multipoint linkage analysis (67%) and more closely approximated defining the theoretical portion of the genome that should be shared or identical by descent among the affected individuals (7.4% by haploid analysis vs. 3.0% theoretical).

### SNP Markers

As a second and parallel approach to maximize the genetic information from this small ALS family, we investigated the utility of high density SNP arrays to predict shared haplotypes. While SNP arrays have typically been used to examine heterogeneous DNA samples in association studies, we sought to determine whether this technology would be successful in large scale haplotype reconstruction in a small family with ethnically similar individuals. By comparing the haplotypes that were experimentally derived using haploid cells lines, we were able to test the accuracy of *in silico* SNP haplotypes predicted by Allegro [22]. Additionally, the SNP haplotypes were subsequently used to validate the power of nonparametric linkage analysis (NPL) of the SNP data to identify shared regions in small kindreds.

### Haplotype Analysis Using SNP Genotypes

#### Comparison of Experimental and In Silico Defined Recombinations

**Figure 5:**
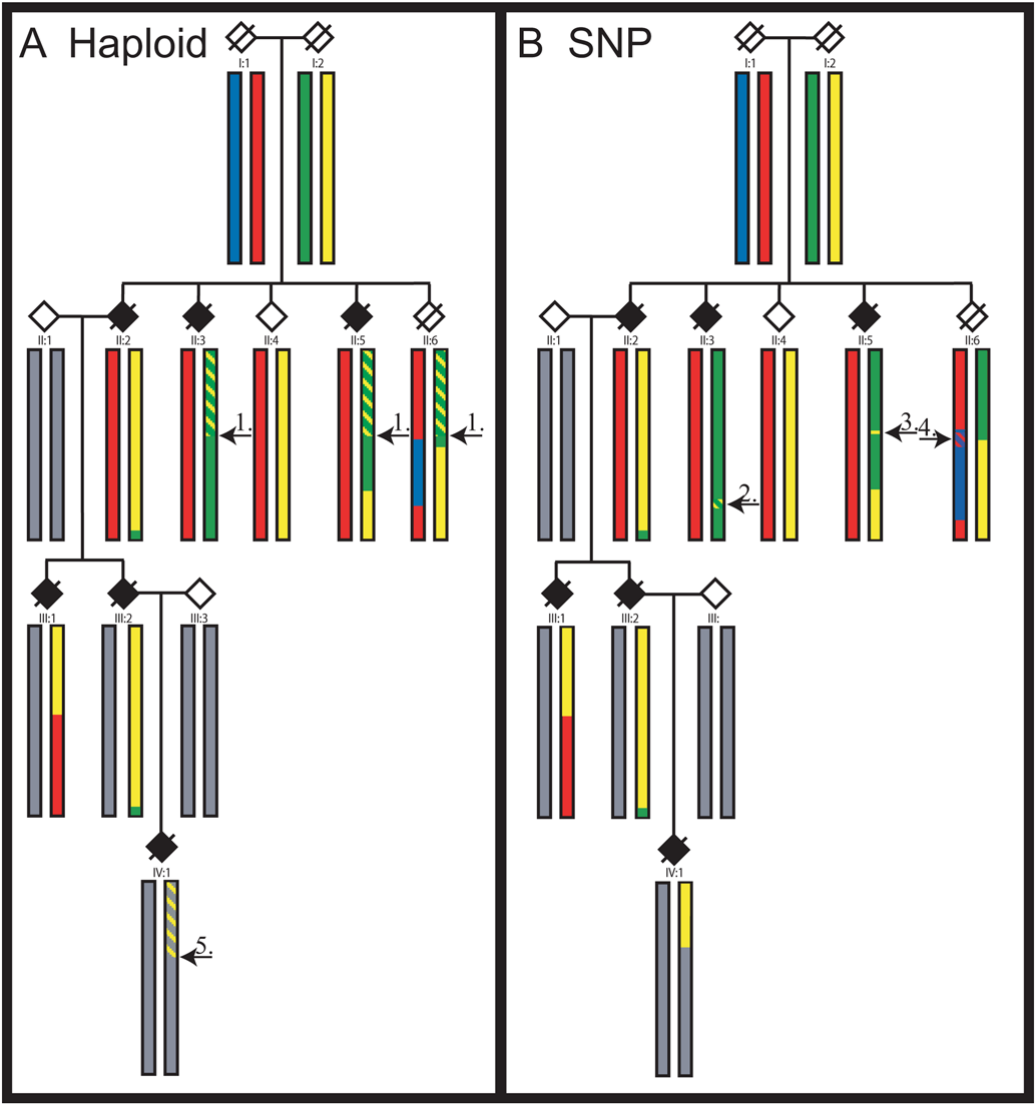
Comparison of Recombination Points Identified by Haploid Mapping and SNPs. Recombinations are depicted for the four founder chromosomes for chromosome 22. The four founder haplotypes are shown in blue, red, green and yellow and the unaffected chromosomes are shown in grey. Arrows point to deviations between the haploid (STRPs) (A) and SNP (B) methods. Regions that are designated by a hatched mixture of two colors result from markers that were not fully informative. The (1) and (5) arrows specify regions where the STRPs were not informative but the SNPs were able to accurately determine the correct haplotype. The (2) and (4) arrows show regions where a block of SNPs were not informative and the haplotypes could not be accurately designated. The (3) arrow represents a region where a double recombination over a small area occurred and was not detected by the STRPs due to marker spacing.

Diploid DNA samples from fourteen members of the ALS-A family were analyzed using the GeneChip™ Human Mapping 100 K Set (Affymetrix) and the resultant data were analyzed using the linkage program Allegro [22]. Specifically, haplotypes were determined *in silico* and were compared with the experimentally defined haplotypes from the haploid cell lines. Evaluation and comparison of recombination points revealed that the SNP arrays were able to precisely and accurately reconstruct haplotypes over large chromosomal regions. Figure 5 shows the comparison of the experimentally and predicted recombinations over the entire length of chromosome 22. While the recombination points are essentially the same, arrows point to deviations between the haploid and SNP methods. Arrows (1) and (5) specify sites where STRP markers were not informative but SNPs accurately defined the haplotype and excluded the regions. Arrows (2) and (4) show regions where a block of SNPs were not informative and the haplotypes could not be unambiguously defined. Arrow (3) represents a region where a double recombination over a small area occurred and was not detected by the STRPs due to marker spacing.

#### Comparison of Experimental and In Silico Haplotypes: Defining Shared Regions

**Figure 6:**
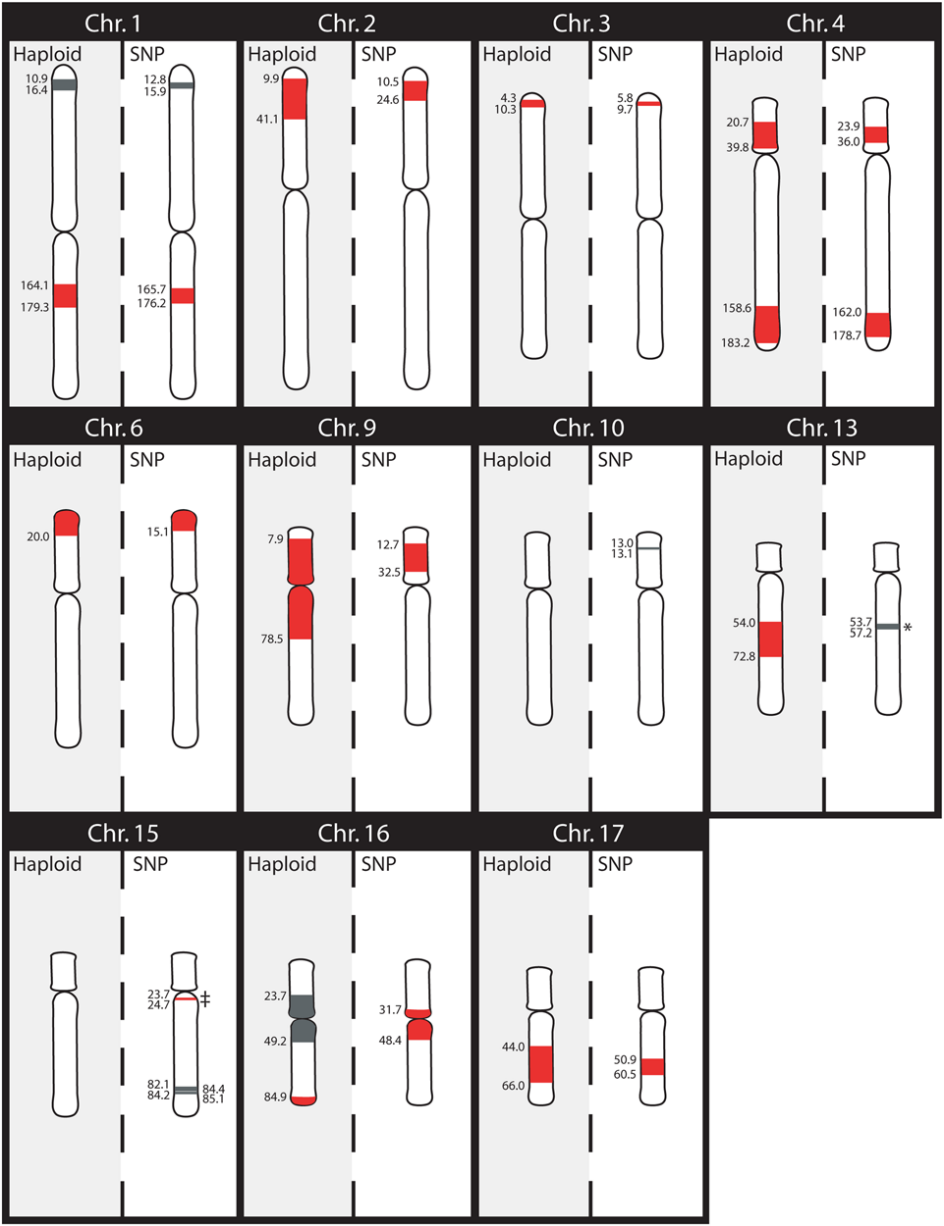
Physical Location of the Shared Regions. The regions that are shared between the five affected individuals are depicted in red. All regions that were identified as ambiguous by the SNP method are shown in grey, along with the any corresponding ambiguous regions identified by the STRP markers. The physical locations of the regions' boundaries were determined by UCSC Genome Browser and approximate positions are shown by each region in Mb. The * indicates the chromosome 13 STRP marker gata73A05, which defines the haploid shared region, is at 62.5 Mb and is not shared by the SNPs at this location. The ‡ symbol denotes an inconsistency between the two methods for the shared region on chromosome 15. While the SNP method identified a shared region, the haploid method identified an ambiguous region with a probability of 1/1000 and was therefore ruled out.

Haplotypes determined from the SNP analysis were examined and regions shared between all five affected individuals were then defined across the entire genome. The SNP method identified a total of 10 shared regions (red), eight of which were detected using the haploid mapping approach and an additional 2 regions that were not previously identified (Figure 6, Table 1). The haploid method was unable to detect the shared region on chromosome 15 because this small interval is located between two STRP markers and on chromosome 16 because two corresponding STRP markers were not informative. Conversely, the SNP method did not detect the shared region on chromosome 13 or the telomeric shared region on chromosome 16 that were identified by the haploid STRP method. A significant advantage of the SNP approach was that each of the shared regions identified were significantly refined and the maximal regions were much smaller than those defined by the haploid STRP approach. Furthermore, the SNP method eliminated nearly all of the ambiguous regions detected by the STRP markers and only detected an additional four new ambiguous regions; the high density of SNP markers significantly cleaned up the data and removed nearly all ambiguity. Nearing the theoretical value of 3%, these methods show that approximately 4.7% (142 megabase pairs (Mb)) of the genome is shared among the five affected individuals. Table 1 lists the shared regions identified by the two methods and the ambiguous regions detected by the SNPs, along with the markers and physical positions of the boundaries for each region.

**Table 1:** Data for the Haploid and SNP Shared Regions and the SNP Ambiguous Regions.

Shared	Method Identified	Chromosomal Region	Beginning Boundary Marker	Physical Location	Ending Boundary Marker	Physical Location
	Haploid	1q24.1-25.3	ATA38A05	164,115,369	AAT200	179,292,171
	SNP	1q24.2-25.2	rs952963	165,669,836	rs10489882	176,171,118
	Haploid	2p25.1-22.1	D2S423	9,858,029	GATA194B06	41,077,763
	SNP	2p25.1-23.3	rs1405948	10,500,611	rs1822300	24,578,289
	Haploid	3p26.2-25.3	GATA131D09	4,288,250	ATCT053	10,338,466
	SNP	3p26.1-25.3	rs2196302	5,780,096	rs3868891	9,674,059
	Haploid	4p15.31-14	GATA70E01	20,722,813	ATCT018	39,814,243
	SNP	4p15.2-p14	rs4697055	23,876,434	rs7695130	35,959,708
	Haploid	4q32.1-35.1	D4S1629	158,556,255	AATA045	183,213,532
	SNP	4q32.2-34.3	rs6811083	162,010,831	rs4234867	178,742,190
	Haploid	6p25.3-p22.3	NA	1	D6S1959	20,020,074
	SNP	6p25.3-23	NA	1	rs2327869	15,096,746
	Haploid	9p24.1-q21.13	D9S2156	7,918,932	AGAT140	78,454,471
	SNP	9p23-21.1	rs10491744	12,710,106	rs621277	32,506,864
	Haploid	13q21.1-22.1	D13S784	54,003,967	D13S800	72,772,650
	SNP	15q12	rs1553890	23,688,680	rs4073083	24,737,820
	SNP	16p11.2-q12.1	rs10492807	31,687,619	rs7500906	48,354,332
	Haploid	16q24.1-24.3	D16S539	84,943,535	NA	88,827,254
	Haploid	17q21.32-24.3	D17S2180	44,028,054	D17S2059	66,012,504
	SNP	17q22-24.1	rs716392	50,944,412	rs10514869	60,531,720
**Ambiguous**	Haploid	1p36.22-p36.13	AAT238	10,910,244	TTTA063	16,412,894
	SNP	1p36.21	rs848578	12,760,221	rs10492987	15,865,925
	SNP	10p13	rs963336	12,964,446	rs552437	13,087,512
	SNP	13q21.1	rs7335975	53,714,107	rs9316943	57,196,412
	SNP	15q25.2-25.3	rs10520582	82,143,780	rs10520601	84,159,205
	SNP	15q25.3	rs2120650	84,419,700	rs4887244	85,147,853
	Haploid	16p12.1-q12.1	CATA002Z	23,656,985	GATA143D05	49,152,042

Physical locations were determined using the UCSC Genome Browser (www.genome.ucsc.edu) and the NCBI SNP database (http://www.ncbi.nlm.nih.gov/sites/entrez?db=snp) was used to convert the SNP ID into the RS nomenclature recognized by the UCSC Browser (March 2006). The STRPs were taken from the Marshfield screening sets 13 and 52.

### Parametric and Non-parametric Linkage Analysis

**Figure 7:**
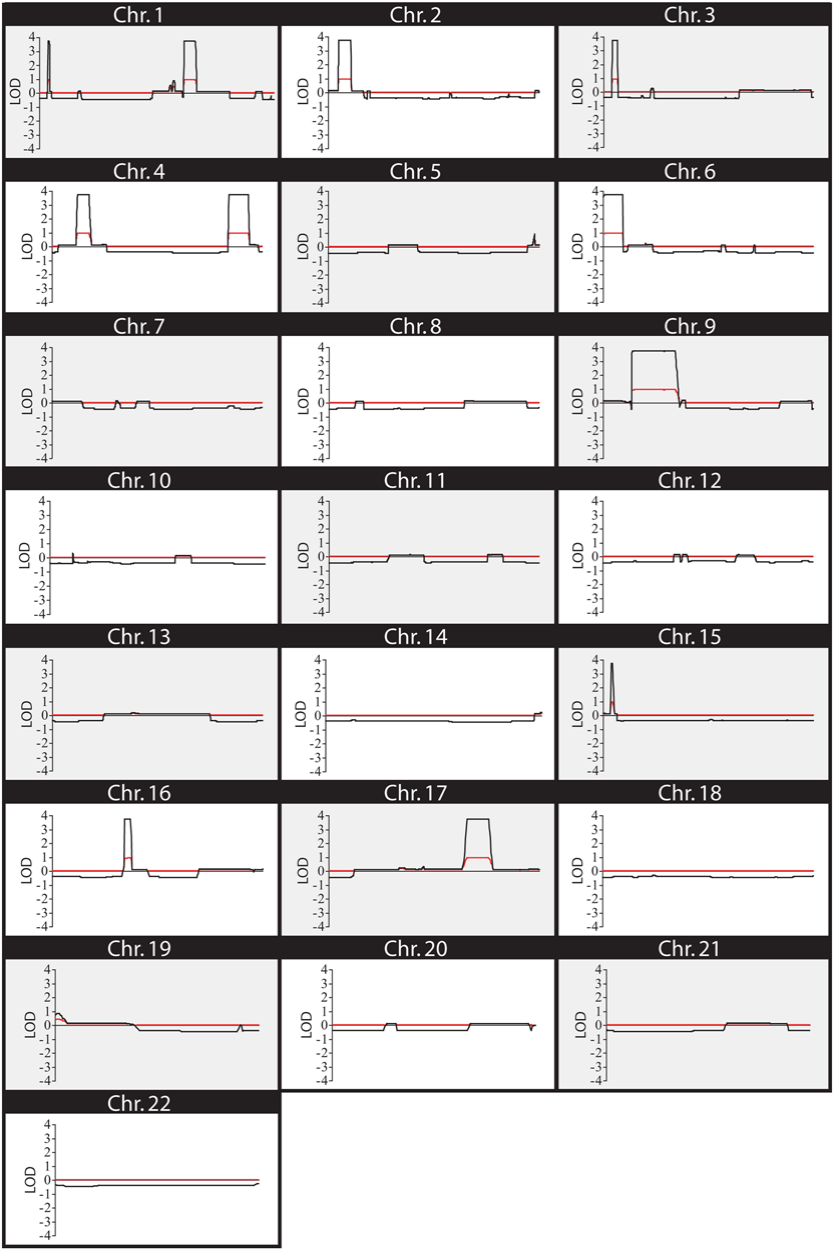
Non-parametric LOD Score Analysis of the SNP Genotypes. The SNP genotype data were analyzed by Allegro and non-parametric LOD scores were generated for each autosomal chromosome. The NPL scores are shown by a black line and the red line represents the allele-sharing LOD. Chromosomal location is shown along the X-axis.

In addition to examining the utility and accuracy of predicted SNP haplotypes, we also investigated the power of nonparametric or model free linkage analysis to detect shared chromosomal regions. The non-parametric linkage analyses (NPL and allele sharing LOD scores), which perform statistical analysis of an increase in the number of alleles shared among the affected individuals with respect to identity by descent (IBD), weigh the genotypes of affected individuals rather than those of unaffected individuals; therefore all shared regions have equally high allele sharing LOD and NPL scores and can be easily identified. Using the linkage program Allegro [22] we generated both NPL and allele sharing LODs for all of the autosomal chromosomes, which are depicted in Figure 7. This method clearly distinguished all ten regions identified by the SNP haplotypes, confirming the power of model free analysis in the identification of candidate regions within small families. Parametric analysis was also performed; because the genotypes from unaffected, but at-risk individuals were factored into the calculations not all of the shared candidate regions were clearly identifiable (Figure S1).

### Prioritization of the Shared Regions

As a final step, additional pedigree analysis was done to prioritize the candidate gene regions to those shared among affected individuals but absent from older unaffected family members. Although the range in disease onset is broad in the ALS-A family (35–73 years), two unaffected elderly individuals, II∶3 and II∶9, had no evidence of the disease; neither had signs or symptoms of ALS when examined (ages 68 and 82, respectively). Additionally, individual II∶3 had no signs of ALS when interviewed at age 76, and prior to dying from Alzheimer's disease at age 90 y, individual II∶9 did not demonstrate ALS symptoms according to relatives or medical records. There was no evidence of frontotemporal dementia in the family, including II∶9, who had onset of dementia in the late 80 s, no unusual behavior while dementing, and responded to anticholinesterase medication. Although neither II∶3 and II∶9 showed signs of ALS, the possibility that they carry/carried the disease gene can not be excluded. However, the age of these individuals make the risk that they carry the ALS-A gene quite low, and hence make the regions of the genome that they do not share with the affected individuals more likely to contain the gene. Similarly, regions shared by all five affected individuals and II∶3 or II∶9 have a lower probability of containing the ALS-A gene. Using this approach we have prioritized the candidate regions into three categories (RI-1-3), with RI-1 being the most likely (Table 2). Three of these regions/subregions, which span ∼23 Mb, are higher priority regions for future studies because they are not shared in older unaffected members of the ALS-A family: the 6p25.3-23 region, ∼50% of the 4p15.2-p14 region and ∼7% of the 4q32.2-34.3 (Table 2). We would predict that ∼23 Mb should be shared by the five affected individuals and not shared by the two unaffected individuals, which matches our experimental results almost exactly. The remaining unaffected individuals within the ALS-A family were not useful in the prioritization because they are still within the disease onset range.

**Table 2:** Genetic Status of Unaffected Individuals II∶3 & II∶9 for the Seventeen Shared and Ambiguous Regions.

Shared	Region of Interest Rank	Region	Maximum Size (Mb)	Unaffected Individual	Unaffected Individual
	RI-1	6p25.3-23	15.1 Mb		
	RI-1/2	4p15.2-p14	12.1 Mb	P (49.2%)	P (6.3%)
	RI-1/2	4q32.2-34.3	16.7 Mb	P (92.4%)	
	RI-2	2p25.1-23.3	14.1 Mb		X
	RI-2	3p26.1-25.3	3.9 Mb	X	
	RI-2	13q21.1-22.1	18.8 Mb		X
	RI-2	15q12	1.0 Mb	X	
	RI-2	16q24.1-24.3	3.9 Mb	X	
	RI-2	17q22-24.1	9.6 Mb		X
	RI-2/3	9p23-21.1	19.8 Mb	A (12.4%)	X
	RI-2/3	16p11.2-q12.1	16.7 Mb	X	P (94.2%)
	RI-3	1q24.2-25.2	10.5 Mb	X	X
**Ambiguous**	RI-3	1p36.21	3.1 Mb	X	X
	RI-3	10p13	0.1 Mb	X	X
	RI-3	13q21.1	3.5 Mb	X	X
	RI-3	15q25.2-25.3	2.0 Mb	X	X
	RI-3	15q25.3	0.7 Mb	X	X

The letter “X” specifies the region is also shared by the unaffected individual, the letter “P” indicates the region is partially shared and the letter “A” designates the region is ambiguous. The maximum physical size is shown for each region. The shared regions of interest are classified into three categories based on the genomic content of the two unaffected individuals (II∶3 and II∶9). The RI-1 category contains regions that are the most likely to contain the ALS-A gene while the RI-3 category includes regions that are the least likely to hold the gene.

## Discussion

In linkage studies of large families with Mendelian disorders LOD score analysis is an effective method to define the disease locus i.e. the single region of the genome that is shared among affected individuals. However, in small families with limited numbers of meioses, traditional LOD score analysis using STRP markers is less informative, often showing suggestive LOD scores for regions of the genome that should be excluded and missing other regions that may contain the gene. We have explored methods to maximize the amount of genetic information that can be derived from a small family with an autosomal dominant form of ALS and show that analysis of high density SNP haplotypes are able to precisely define nearly all regions of the genome that are shared among the affected individuals. Specifically, we used haploid cell lines to experimentally demonstrate that high density SNP arrays can be used to accurately define chromosomal haplotypes, precisely identify shared candidate regions among affecteds and exclude the remaining regions of genome that are not shared. Additionally, we show that SNP genotypes can be accurately analyzed by nonparametric linkage programs and validate the power of model free analysis to detect shared regions in small families.

Over the past 20 years, genetic investigations using families with monogenic disorders have provided unparalleled information into gene function, normal and pathogenic pathways and disease mechanisms [1]. Because most of the remaining single-gene families have rare mutations and/or few affected members, new strategies are needed to effectively use this valuable resource for genetic studies. Shifting our expectations from the idea that mapping single-gene disorders necessarily requires sufficient meioses to localize the gene to a single site in the genome, to the idea that small families can also be included in genetic studies if as a first step all shared regions of the genome can be accurately defined. Because novel disease genes in small families are likely to hold key lessons for understanding the molecular pathophysiology of devastating disorders, it is imperative that these families are investigated and that candidate loci are reported—informed collaborative efforts will lead to the eventual identification of these genes.

Towards this effort, we have mapped a rare familial form of ALS that is clinically similar to sALS. Candidate regions for the ALS-A gene span 142 Mb of DNA located in twelve intervals. Three of these regions/subregions, which span ∼23 Mb, are higher priority regions for future studies because they are not shared in older unaffected members of the ALS-A family. While the following paragraphs describe the next steps in identifying the ALS-A gene, similar strategies would be generally applicable to mapping efforts of any single gene disorder in any similarly small family. First, newly identified candidate regions can be refined and prioritized, additional families with similar diseases can be screened for linkage to separate candidate loci and genes of interest can be sequenced.

Comparisons of known ALS loci with our newly defined candidate region show several regions of overlap or possible overlap. For example, an ambiguous region defined by the haploid cell lines at 1p36.21 contains the recently identified *TARDBP* gene. Mutations in this gene cause a phenocopy of sALS [17], however, this gene has been ruled out by the SNP haplotype analysis and sequencing of an affected member of the ALS-A family. Additionally, our initial mapping showed potential overlap with the ALS6 gene, which was recently shown to be caused by mutations in FUS/TLS on 16p11.2 [18], [19]. Although ALLEGRO predicted a recombination that would rule out FUS/TLS, a detailed inspection of the 100 K SNP data and genotyping analysis of additional SNPs in the region showed that FUS/TLS lies within a region between rs7193224 and rs2141349 where a key affected recombinant has the unaffected haplotype at rs7193224 and the affected haplotype at rs2141349, a marker lying very close to FUS/TLS. Therefore, the FUS/TLS locus cannot be conclusively excluded from the SNP analysis. Subsequent sequence analyses of the exons and intron/exon boundaries from an affected individual found no causative mutations. Additionally, one of the 12 candidate regions overlaps with the ALS-FTD locus on 9p21.3-13.2 [23]–[25].

The disease spectrum for both ALS and FTD are still evolving, and families linked to the ALS-FTD locus on chromosome 9 have members that present with pure ALS, pure FTD or both [23]–[25]. Therefore, while members of the ALS-A family have not displayed symptoms of FTD, this shared region remains a viable candidate. However, because this region is shared by one of the two unaffected individuals in generation II (Table 2), the likelihood that this region contains the mutation is low.

To further refine the ALS-A locus we propose additional independent linkage studies using other small ALS families with clinically similar forms of ALS. While many of these additional families will be even smaller than the ALS-A family, our mapping study will enable other groups to determine if ALS families they have collected share any of the candidate ALS-A regions we describe here. Multiple families with linkage to one of these regions would provide additional support for prioritizing specific regions for detailed gene cloning efforts. Additionally, haplotype conservation could indicate the presence of an ancestral mutation and potentially pinpoint regions of special interest for sequencing.

As a complementary approach, we have begun examining candidate genes within shared regions, initially focusing on genes encoding proteins expressed within the CNS or that act in pathways implicated in ALS. For example, we sequenced *superoxide dismutase 3* (*SOD3*) located within the 4p15.2-p14 region, due to the similarity with *SOD1*, although no mutations were detected in the exons, exon/intron boundaries or upstream sequence. While careful examination of known genes will not be as complicated in small gene poor areas (chr.4p), this process will be more difficult in large gene rich regions, such as the shared interval on chromosome 6p. Therefore, in addition to targeted gene sequencing, brute force sequencing of entire shared regions may be appropriate.

In summary, we have demonstrated through experimentally derived haplotypes using haploid DNA that dense genomic SNP arrays can accurately define chromosomal haplotypes in small families. Additionally, we experimentally validate the power and effectiveness of model free linkage analysis of SNP genotypes in the detection of the shared candidate regions. Application of this rapidly improving technology will enable genetic investigations of a whole class of families with Mendelian disorders that have typically been ignored by the scientific community due to their size. By changing the mapping paradigm from the idea that families with Mendelian disorders need to be large enough to map the gene to a single site in the genome, to include the concept that small families can be useful if all regions that could contain the gene are identified, we can begin to use a valuable and virtually untapped resource. Improving the power of mapping single-gene disorders will uncover new disease pathways and mechanisms and clarify the pathogenesis of devastating disorders like ALS.

## Materials and Methods

### Ethics Statement

This study was conducted according to the principles expressed in the Declaration of Helsinki. All subjects participating in this study signed an informed consent form approved by the Human Subjects Committee at the University of Minnesota. DNA was extracted from peripheral venous blood using the Gentra Puregene blood kit (Qiagen, Valencia, CA).

### Generation of haploid cells lines

Panels of haploid mouse/human hybrid cell lines were generated at GMP Genetics for eight ALS-A family members (Figure 1) by electrofusing lymphoblast cells from each individual with embryonic day 2 (E2) mouse cells, using HAT and geneticin to select against unfused E2 and lymphoblast cells, respectively [20], [26]. Eighteen to thirty seven cell lines were selected for each individual. In general, each hybrid cell line should contain 8–14 random human chromosomes, some of which are in the haploid state while others are diploid. On average, an entire single set of monosomic chromosomes is represented in ∼23 independent cell lines [26], [27].

### 800 Marker Genome Screen

A 777 STRP marker high-density genome screen was performed on both diploid and haploid DNA from members of the ALS-A family using fluorescent technology (Figure 1). The work was performed by the Center of Medical Genetics, Marshfield, WI as part of the NHLBI Mammalian Genotyping Service (Contract Number NO1-HV-48141). Markers were taken from Marshfield screening sets 13 and 52 and all of the cell lines generated by GMP Genetics were included in the genome screen. Amplified DNA from parents of CEPH family 1331 (133101 and 133102) were used as standards for all test samples.

### Haplotype Ascertainment

Haplotypes for each individual were directly determined along entire chromosomes by analyzing the genotypes of cell lines haploid for each chromosome, including the 22 autosomes and the X chromosome. The diploid DNA was used as an internal control for each patient and cell lines monosomic for each chromosome were selected for further analysis so that each homologue was represented. For the eight patients in which haploid cell lines were established, phase was determined for each chromosome using the two independently represented homologues. However, rarely both homologues were not present in the monosomic state, in which case haplotypes were defined by comparing genotypes of the available haploid cell line with the diploid DNA.

### Multipoint LOD Score Analysis

Linkage analysis of the 22 autosomes and the X chromosome were performed using the computer program VITESSE version 2.0 [21]. Five age-dependent penetrance classes were established for at-risk unaffected individuals based on the age-at-onset profile for the family (<20 years, 5%; 20–30 years, 10%; 31–60 years, 50%; 61–70 years, 80%; 71+ years, 90%). Affected individuals and unaffected spouses were classified separately. The lifetime risk of developing a phenotypically similar disease, sALS, was estimated to be 1/1000. Allele frequencies and the order of the microsatellite markers used in the genome screen were established by Marshfield Genetics.

### 100 K SNP Screen and analysis

High-density SNP based linkage analysis was conducted using the GeneChip™ Human Mapping 100 K Set (Affymetrix, Santa Clara, CA). SNP genotype data were extracted with the following “cut-off values”: call rate of control samples >0.95, p value of HWE (Hardy-Weinberg equilibrium test) in control samples >0.05, MAF (minor allele frequencies) of control samples >0, and confidence scores of all family members <0.1. Control samples were collected from 24 healthy Japanese individuals. SNPs were selected to keep the inter-marker distance at approximately 100 kb. Parametric and non-parametric multipoint linkage analyses were performed using the Allegro version 2.0 program [22]. Penetrance classes were set as described above, and disease gene frequencies were set at 0.001. In the non-parametric analysis the following allele sharing model was used: multipoint, linear model, robdom and power = 0.5. Because the size of inheritance vectors of this family exceeded the limitation of 21, we calculated LOD scores in which two unaffected individuals (III∶1 and III∶2) were removed. Haplotype prediction was performed using the ‘haplotype’ option in the multipoint linkage analysis of the Allegro version 2.0 package.

## Supporting Information

Figure S1 Parametric LOD Score Analysis of the SNP Genotypes. Parametric LOD Score Analysis of the SNP Genotypes. The SNP genotype data were analyzed by Allegro and parametric LOD scores were generated for each autosomal chromosome. Chromosomal location is shown along the X-axis.
(2.38 MB EPS)
